# Supplementary material for: The interactome of intact mitochondria by cross-linking mass spectrometry provides evidence for coexisting respiratory supercomplexes
Source: Mol Cell Proteomics. 2017 Dec 8;17(2):216–32. doi: 10.1074/mcp.RA117.000470 (PMC5795388; doi:10.1074/mcp.RA117.000470)
Supplement: Supplemental Data [file supp_RA117.000470_133922_0_supp_23978_fzffwf.zip › spectra_annotation/mito_spectra_annotation/500-1-11-1-6-1.pdf]

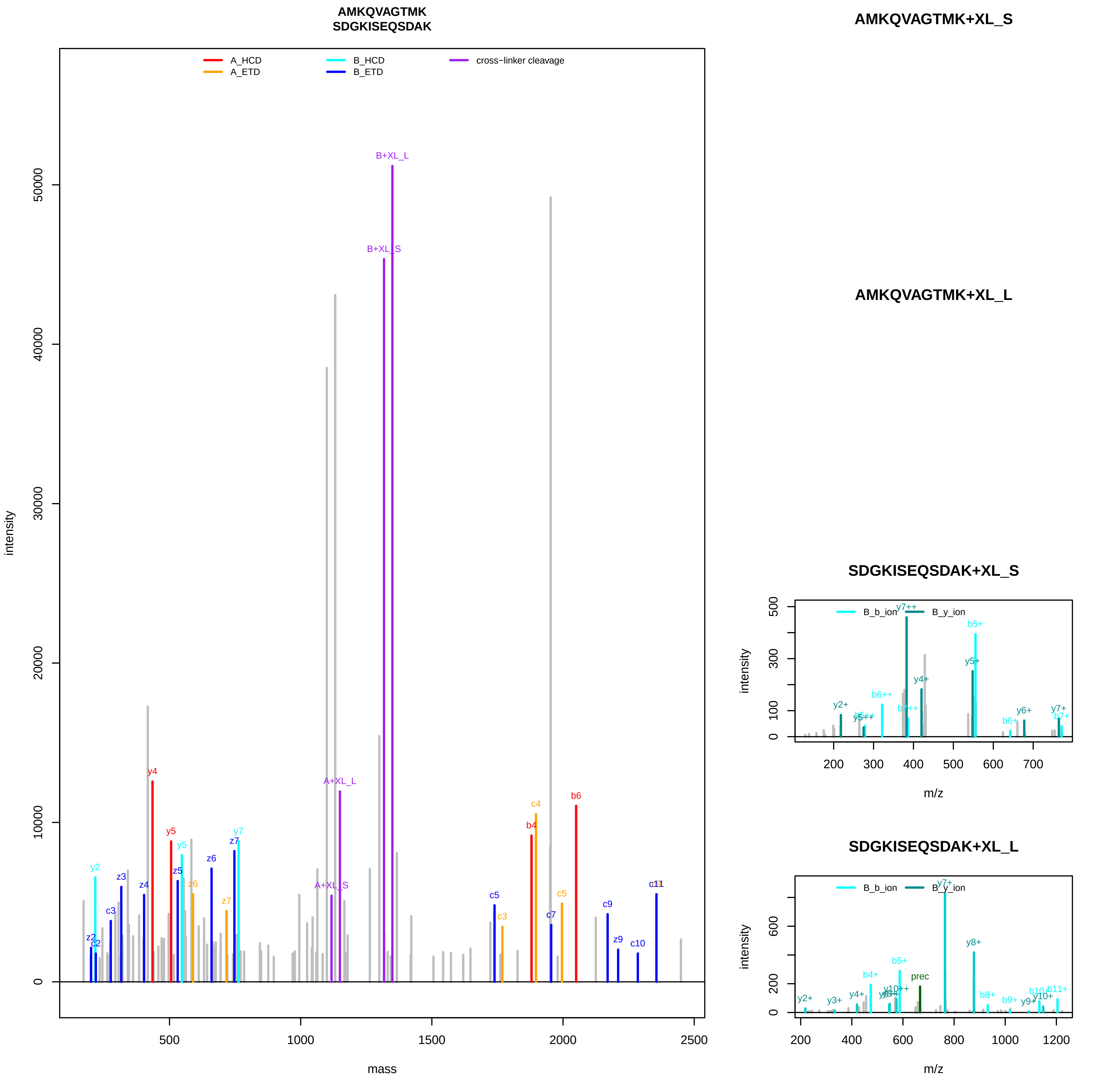
AMKQVAGTMK
SDGKISEQSDAK
A_HCD
A_ETD
B_HCD
B_ETD
cross−linker cleavage
intensity
mass
0
10000
20000
30000
40000
50000
500
1000
1500
2000
2500
B+XL_L
B+XL_S
A+XL_L
A+XL_S
y2
z2
c2
c3
z3
z4
y4
y5
z5
y5
z6
z6
z7
z7
y7
c5
c3
c4
b4
c7
c5
b6
c9
z9
c10
c11
AMKQVAGTMK+XL_S
AMKQVAGTMK+XL_L
SDGKISEQSDAK+XL_S
B_b_ion
B_y_ion
intensity
m/z
0
100
300
500
200
300
400
500
600
700
y7++
b5+
y5+
y4+
y2+
b6++
y6+
y7+
b7+
b6+
SDGKISEQSDAK+XL_L
B_b_ion
B_y_ion
intensity
m/z
0
200
600
200
400
600
800
1000
1200
y7+
y8+
b5+
b4+
prec
y2+
y3+
y4+
y10++
b8+
b9+
y9+
y10+
b11+
